# Recombinant *Saccharomyces cerevisiae* EBY100/pYD1-FaeG: a candidate for an oral subunit vaccine against F4+ ETEC infection

**DOI:** 10.1128/aem.01817-24

**Published:** 2024-11-27

**Authors:** Dayue Hu, Xiangmin Li, Xiaochao Duan, Liuyue Yang, Baizhi Luo, Linkang Wang, Zihui Hu, Yang Zhou, Ping Qian

**Affiliations:** 1 National Key Laboratory of Agricultural Microbiology, Hubei Hongshan Laboratory, Huazhong Agricultural University 47895 , Wuhan, China; 2 The Cooperative Innovation Centre for Sustainable Pig Production, Huazhong Agricultural University 47895 , Wuhan, China; 3 College of Veterinary Medicine, Huazhong Agricultural University 47895 , Wuhan, China; 4 College of Life Science and Technology, Huazhong Agricultural University 47895 , Wuhan, China; Centers for Disease Control and Prevention, Atlanta, Georgia, USA

**Keywords:** F4+ ETEC, oral vaccine, *Saccharomyces cerevisiae*

## Abstract

Diarrheal diseases attributable to multidrug-resistant F4+ enterotoxigenic *Escherichia coli* (ETEC) are escalating in severity, posing significant risks to the health and safety of both humans and animals. This study used *Saccharomyces cerevisiae* EBY100 to display the FaeG subunit of F4 colonizing factor as an oral vaccine against F4+ ETEC infection. Mice were orally immunized twice with 10^8^ CFU of EBY100/pYD1-FaeG, followed by a challenge with F4+ ETEC EC6 on day 7 post-immunization. The results showed that the recombinant strain EBY100/pYD1-FaeG orally enhanced the growth of the small intestine villi, significantly boosted the expression of tight junction proteins (*ZO-1*, *Occludin*, *MUC2*, and *Claudin*) (*P* < 0.05), and modulated the gut microbiota composition. Additionally, immunization with EBY100/pYD1-FaeG also upregulated the levels of IL-2, IL-4, and IFN-γ in the intestines of mice (*P* < 0.01), while serum IgG and fecal sIgA titer significantly increased (*P* < 0.05). These immune responses enhanced the capacity to fight against ETEC, leading to an increased survival rate of mice and relieved damage to tissues and organs of mice infection. In summary, the study suggested that the recombinant *Saccharomyces cerevisiae* EBY100/pYD1-FaeG could effectively stimulate the immune response and generate specific antibodies against F4+ ETEC, showing its potential to serve as a subunit oral vaccine candidate for preventing F4+ ETEC infection.

**IMPORTANCE:**

The multidrug-resistant F4+ enterotoxigenic *Escherichia coli* (ETEC) strains are the primary clinical pathogens responsible for post-weaning diarrhea in piglets, resulting in substantial economic losses in the pig farming industry. In the study, we developed an oral vaccine candidate, *Saccharomyces cerevisiae* EBY100/pYD1-FaeG, to prevent diarrhea caused by multidrug-resistant F4+ ETEC. Oral administration of EBY100/pYD1-FaeG significantly enhanced immune responses, improved intestinal health, and provided protection against F4+ ETEC infection in mice. This approach offers a potential application prospect for preventing F4+ ETEC infections that lead to post-weaning diarrhea in clinical settings and provides a promising solution for addressing the growing threat of antibiotic resistance in bacterial pathogens.

## INTRODUCTION

Enterotoxigenic *Escherichia coli* (ETEC) is a common zoonotic and foodborne pathogen responsible for causing diarrhea in both humans and juvenile animals (1, 2). In addition, ETEC can also cause post-weaning diarrhea in piglets (PWD) (3, 4). ETEC mainly produces two virulence factors: adhesins, which promote bacterial colonization, and enterotoxins, which are implicated in the regulation of fluid secretion (1). The adhesion of ETEC is an important link in its pathogenesis. It promotes bacteria to bind to specific intestinal epithelial cell receptors for intestinal colonization (5). Once ETEC adhesion is established in the small intestines of animals, ETEC will create heat-stable enterotoxins or heat-labile enterotoxins. These toxins induce diarrhea, which will cause the death of ETEC-infected newborns owing to severe watery diarrhea, severe dehydration, and electrolyte imbalance (6). The adhesins of ETEC are expressed in fimbriae, with F4, F5, F6, F18, and F41 being the typical fimbriae (7, 8). ETEC strains that cause newborn diarrhea are linked to fimbriae F5, F6, and F41, whereas strains associated with PWD typically express fimbriae F18 (8–10). Additionally, fimbriae F4 are connected to strains that cause both neonatal diarrhea and PWD (3, 11, 12). The FaeG subunit is important for F4+ ETEC strain binding to intestinal epithelial cell receptors and colonization and results in a pathogenic weakening of the strain (13–15).

With the global ban on zinc oxide and feeding antibiotics, the cases of newborn diarrhea and PWD will continue to rise. The occurrence of PWD has brought considerable economic losses to the global pig industry (16–19). The rising crisis of drug resistance presents a serious threat to the health and well-being of both animals and humans, as well as to public health safety. Finding safe and effective alternatives to antibiotics has become a global research hotspot.

*Saccharomyces cerevisiae* (*S. cerevisiae*) is a unicellular eukaryotic organism with good biosafety, a clear genetic background, and GRAS status (20, 21). As a probiotic, *S. cerevisiae* is widely used in medical treatment, food, health care, and animal husbandry instead of antibiotics (22). Furthermore, yeast cell wall contains β-1,3-glucan and β-1,6-glucan, which function as natural immune adjuvants capable of stimulating or modulating immune responses (23). *S. cerevisiae* has the capacity to interact with intestinal epithelial cells, thereby inducing mucosal immune responses and regulating intestinal microbiota, which contributes to an increase in the diversity of intestinal flora (24). Many studies have shown that surface-displayed vaccine candidates using *S. cerevisiae* as a carrier showed great promise in preventing bacterial or viral infections (25). For example, Lei et al. (26) used *S. cerevisiae* as a carrier to display H7N9 HA on the surface, which could protect mice 100% from homologous virus challenge. The oral vaccine of *S. cerevisiae* expressing FAdV-4 Fiber-2, prepared by Cao et al. (27), provided good protection to chickens against FAdV-4 infection. In addition, antiviral vaccines such as LMBV, PEDV, SARS-CoV-2, CyHV-2, and ASFV expressed in *S. cerevisiae* have also shown good immune protective effects (28–32).

In this study, the FaeG gene was cloned and expressed to construct a recombinant *S. cerevisiae* strain, EBY100/pYD1-FaeG, for the preparation of an oral vaccine. The immunoprotective efficacy of the EBY100/pYD1-FaeG oral vaccine was assessed *in vivo* using a murine model after confirmation of the expression of the gene *in vitro*. This approach aims to offer a safer, environmentally friendly, and cost-effective strategy for preventing and treating diarrhea induced by F4+ ETEC in clinical practice.

## RESULTS

### Identification of the recombinant *S. cerevisiae* strain expressing the FaeG protein

**Fig 1:**
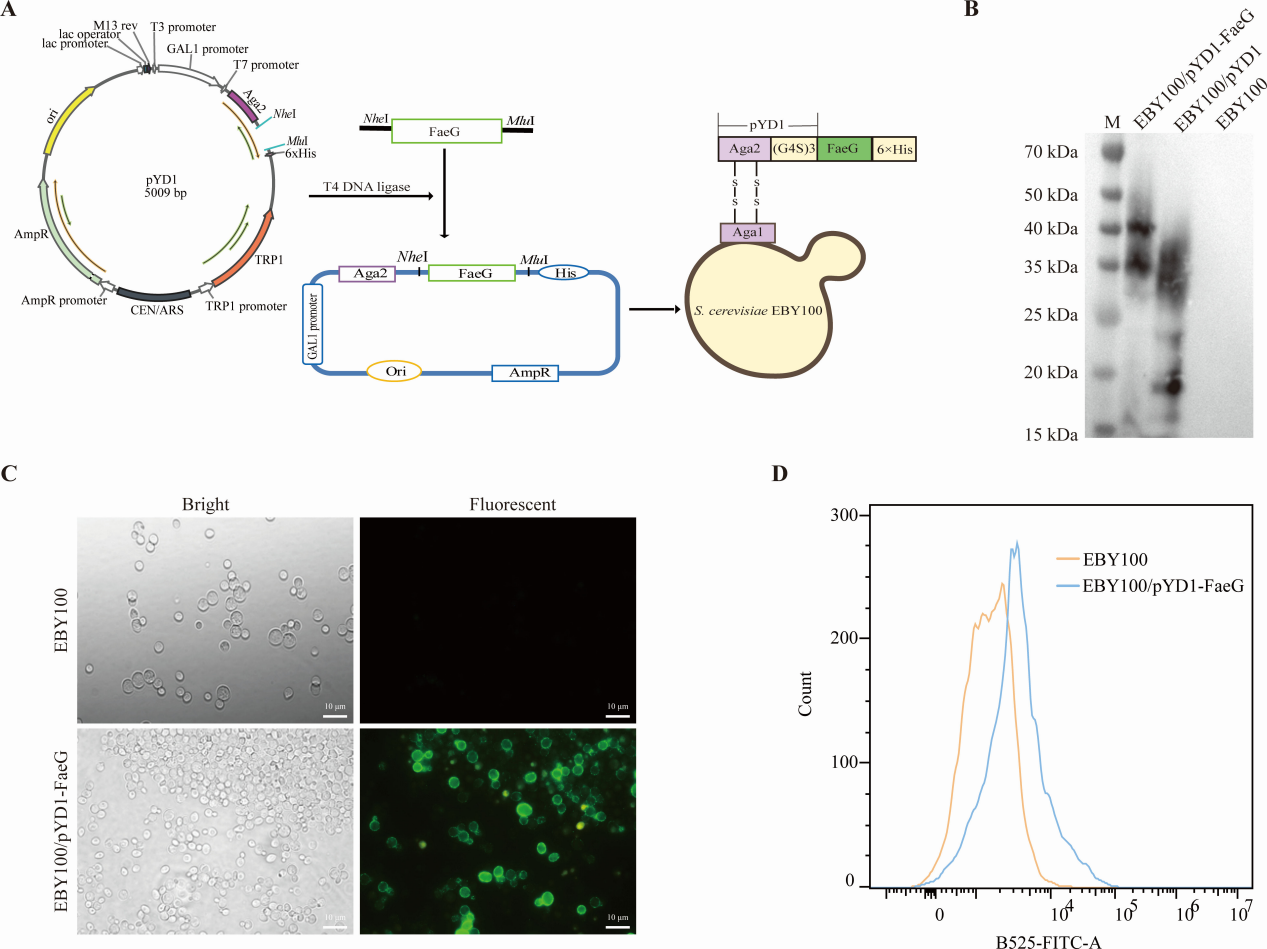
Construction and expression of the recombinant *S. cerevisiae* EBY100/pYD1-FaeG. (**A**) Diagram of the workflow for constructing recombinant yeast. After the FaeG fragment was digested with *Mlu*I and *Nhe*I, the purified FaeG fragment was ligated with T4 ligase with plasmid pYD1. Finally, the recombinant plasmid pYD1-FaeG was transformed into *S. cerevisiae* EBY100 strain by the LiAc method, and positive clones were screened by the minimal dextrose plates. (**B**) Analysis of the expression of the FaeG protein in *S. cerevisiae* EBY100 via western blot. From left to right lanes are M (Protein Maker), EBY100/pYD1-FaeG, EBY100/pYD1, and EBY100. (**C**) The morphology of recombinant *S. cerevisiae* EBY100/pYD1-FaeG under a positive fluorescence microscope. The bar represents 10 µm. (**D**) The expression profile of recombinant protein was also evaluated through flow cytometry.

In this study, the FaeG gene of F4+ ETEC adhesin subunit was inserted into the plasmid pYD1 and linked with Aga2 via (GGGGS)3 linker. In addition, Aga2 was bound to Aga1 through two disulfide bonds to anchor the surface of *S. cerevisiae* EBY100 (Fig. 1A). The expression of the FaeG protein in the recombinant EBY100/pYD1-FaeG strain was detected using western blot analysis. As illustrated in Fig. 1B, the recombinant protein from EBY100/pYD1-FaeG exhibited a band of approximately 42 kDa corresponding to the anticipated molecular weight, while the band for EBY100/pYD1 was approximately 19 kDa, and the lane for EBY100 did not show a specific band. Moreover, the expression of the recombinant protein on the surface of EBY100/pYD1-FaeG strain was further assessed using the immunofluorescence assay (IFA) and flow cytometry. The EBY100 was the negative control. The EBY100/pYD1-FaeG exhibited green fluorescence under the fluorescence microscope (Fig. 1C). The flora of EBY100/pYD1-FaeG showed a significant shift to the right compared with EBY100 in flow cytometry analysis (Fig. 1D). These results demonstrated that the recombinant FaeG protein was efficiently expressed in the strain EBY100/pYD1-FaeG.

### Optimization of growth and expression kinetics of EBY100/pYD1-FaeG strain

**Fig 2:**
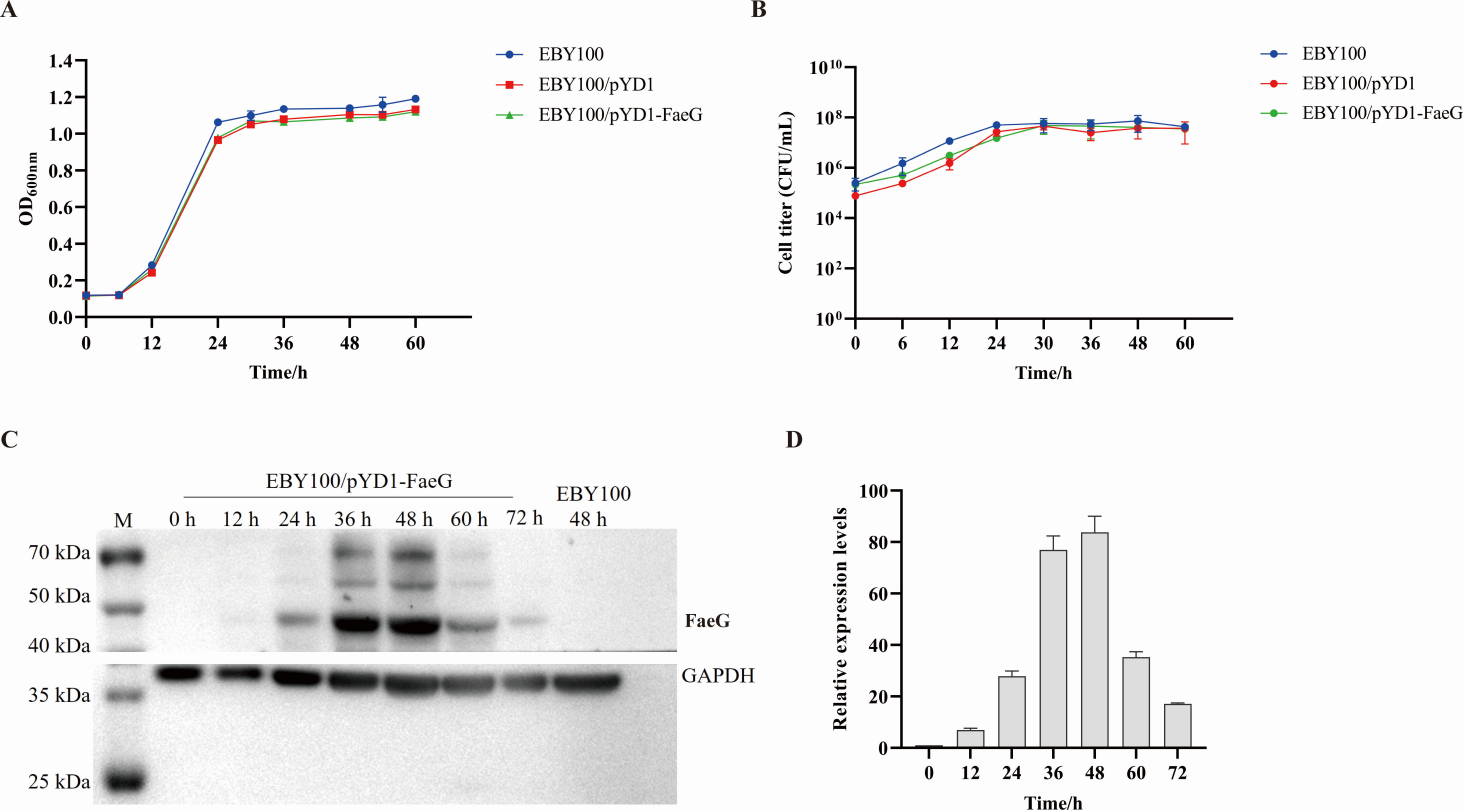
The fermentation and expression kinetics of the FaeG protein in EBY100/pYD1-FaeG. (**A**) The growth curve of the *S. cerevisiae* strains. The OD_600nm_ value of EBY100, EBY100/pYD1, and EBY100/pYD1-FaeG in YPD medium with galactose was measured at various time points. (**B**) The number of cells in YPD medium containing galactose was detected using the plate counting method at the same time points as the OD_600nm_ value measured for the growth curve. (**C**) The expression levels of the FaeG protein at 12, 24, 36, 48, 60, and 72 h in EBY100/pYD1-FaeG were determined by western blotting, with GAPDH as the internal control and the EBY100 strain as the negative control. (**D**) The relative gray value of protein bands was analyzed using the Image J software. All experiments were repeated three times. The data represent the mean ± SD.

To investigate whether the FaeG protein expression affects the growth of the recombinant strain EBY100/pYD1-FaeG, the OD_600nm_ absorbance and colony count of the EBY100, EBY100/pYD1, and EBY100/pYD1-FaeG strains were measured at different growth times in the YPD medium to plot the growth curves. The findings indicated that the growth curves of the recombinant strains of EBY100/pYD1 and EBY100/pYD1-FaeG presented a similar trend (Fig. 2A). Although the OD_600nm_ absorbance of the recombinant strains was observed to be lower than that of *S. cerevisiae* EBY100 during both the logarithmic and stationary phases, the difference was not significant (*P* > 0.05). Concurrently, the corresponding cell count data indicated that the number of cells in the recombinant strains was slightly less than in EBY100 without a statistically significant difference (*P* > 0.05, Fig. 2B). These findings suggested that the expression of the exogenous protein FaeG had no obvious influence on the growth of the recombinant *S. cerevisiae* strain.

In the EBY100/pYD1-FaeG strain, the expression of the FaeG protein at different induction times was examined via western blotting, and the relative gray values of the protein bands were quantitatively analyzed using the Image J software. The results indicated that the expression level of the FaeG protein was relatively higher at 48 h, and it showed a decreasing trend with the extension of expression time (Fig. 2C and D). These results suggested that when the recombinant strain EBY100/pYD1-FaeG was induced with an initial concentration of 0.5 OD_600nm_ at 20°C with shaking at 250 rpm, the optimal induction time for protein expression was 48 h.

### The effects of the recombinant oral vaccine in mice

**Fig 3:**
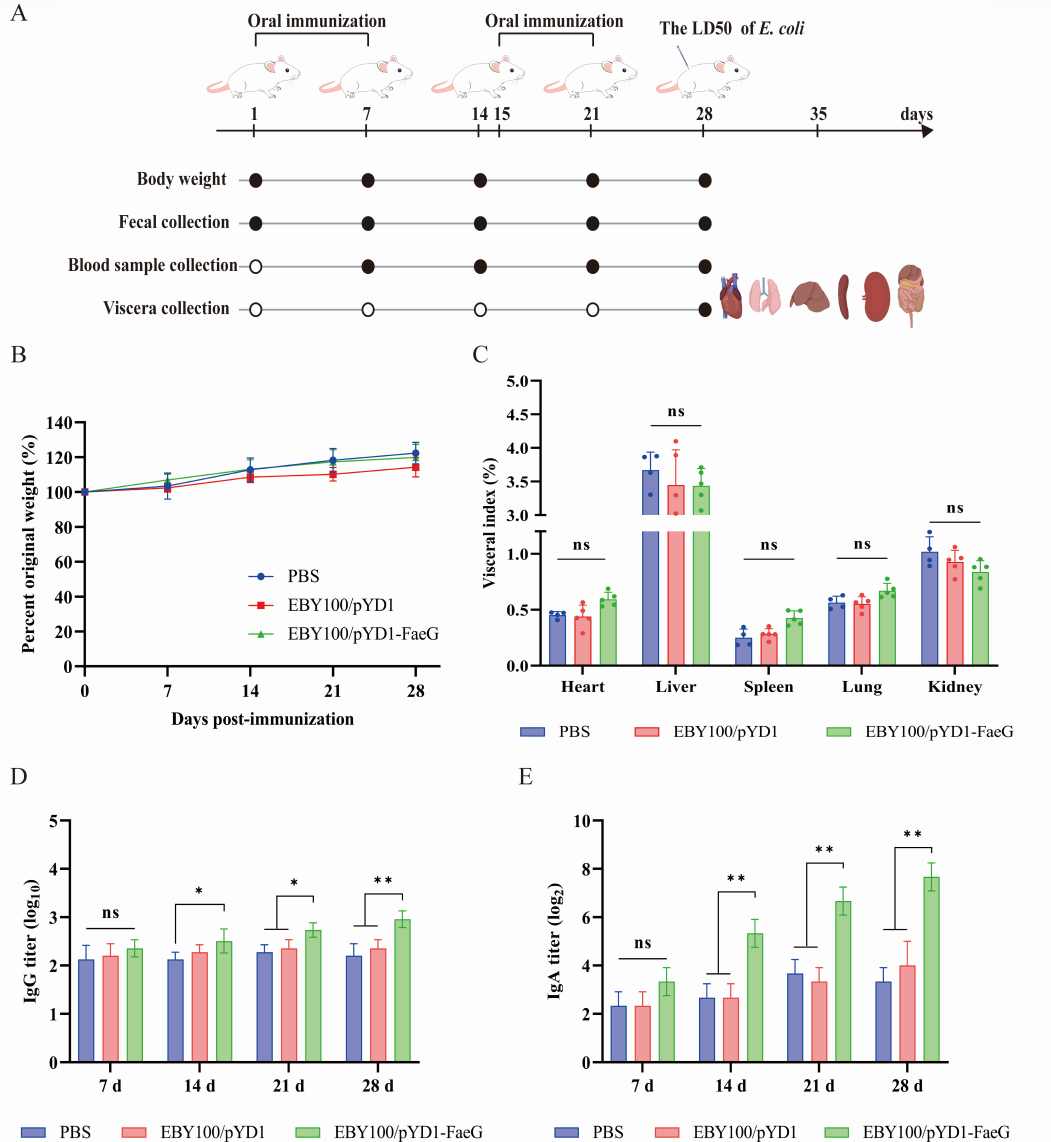
Immune effect of the EBY100/pYD1-FaeG strain on mice. (**A**) Immunization process. Forty-five mice were randomly divided into three groups (15 per group) and were orally immunized twice on days 1–7 and 15–21, respectively. The corresponding samples were collected at different time points (black solid circles), as illustrated in the diagram. (**B**) The changes in body weight in mice at 0, 7, 14, 21, and 28 days (*n* = 10). (**C**) The visceral index of mice on day 28 after immunization. The wet weight to body weight ratio of the heart, liver, spleen, lungs, and kidneys (*n* = 5). (**D**) The specific IgG antibody titers of FaeG protein in the sera of mice after immunization. (**E**) Antibody titers of secretory IgA in feces of mice at days 7, 14, 21, and 28 after immunization. All experiments were performed in triplicate. The data in the figure represent the mean ± SD, and the significance of the difference between groups was assessed via the two-way ANOVA (*P < 0.05; **P < 0.01; ns, no significant difference).

According to the immunization procedure shown in Fig. 3A, 200 μL of the recombinant strain EBY100/pYD1-FaeG was used to immunize mice orally twice on days 1–7 and 15–21, and the PBS control group and the recombinant strain of the empty plasmid group were immunized. To evaluate the effect of EBY100/pYD1-FaeG on the growth of mice, the body weight of mice was recorded on days 7, 14, 21, and 28. As shown in Fig. 3B, the body weight of mice in each group generally increased, and the index of heart, liver, spleen, lungs, and kidneys in each group of mice were similar, without significant differences (*P* > 0.05, Fig. 3C). These findings implied that the recombinant strain EBY100/pYD1-FaeG did not cause adverse reaction in the mouse.

### The recombinant vaccine induced specific antibodies in mice

The OD_450nm_ values for IgG in serum and IgA in fecal samples were measured via ELISA on days 7, 14, 21, and 28 after the initial immunization to evaluate the effectiveness of the humoral and mucosal immune responses induced by EBY100/pYD1-FaeG. As shown in Fig. 3D, the specific IgG antibody titers induced by EBY100/pYD1-FaeG were significantly higher than those in both the EBY100/pYD1 and PBS groups (*P* < 0.05). Meanwhile, specific IgA antibody titers were assessed in the EBY100/pYD1-FaeG group. These titers were also significantly elevated compared to the EBY100/pYD1 and PBS groups on days 14, 21, and 28 (*P* < 0.05, Fig. 3E).

### The recombinant oral vaccine enhanced intestinal mucosal immunity in mice

**Fig 4:**
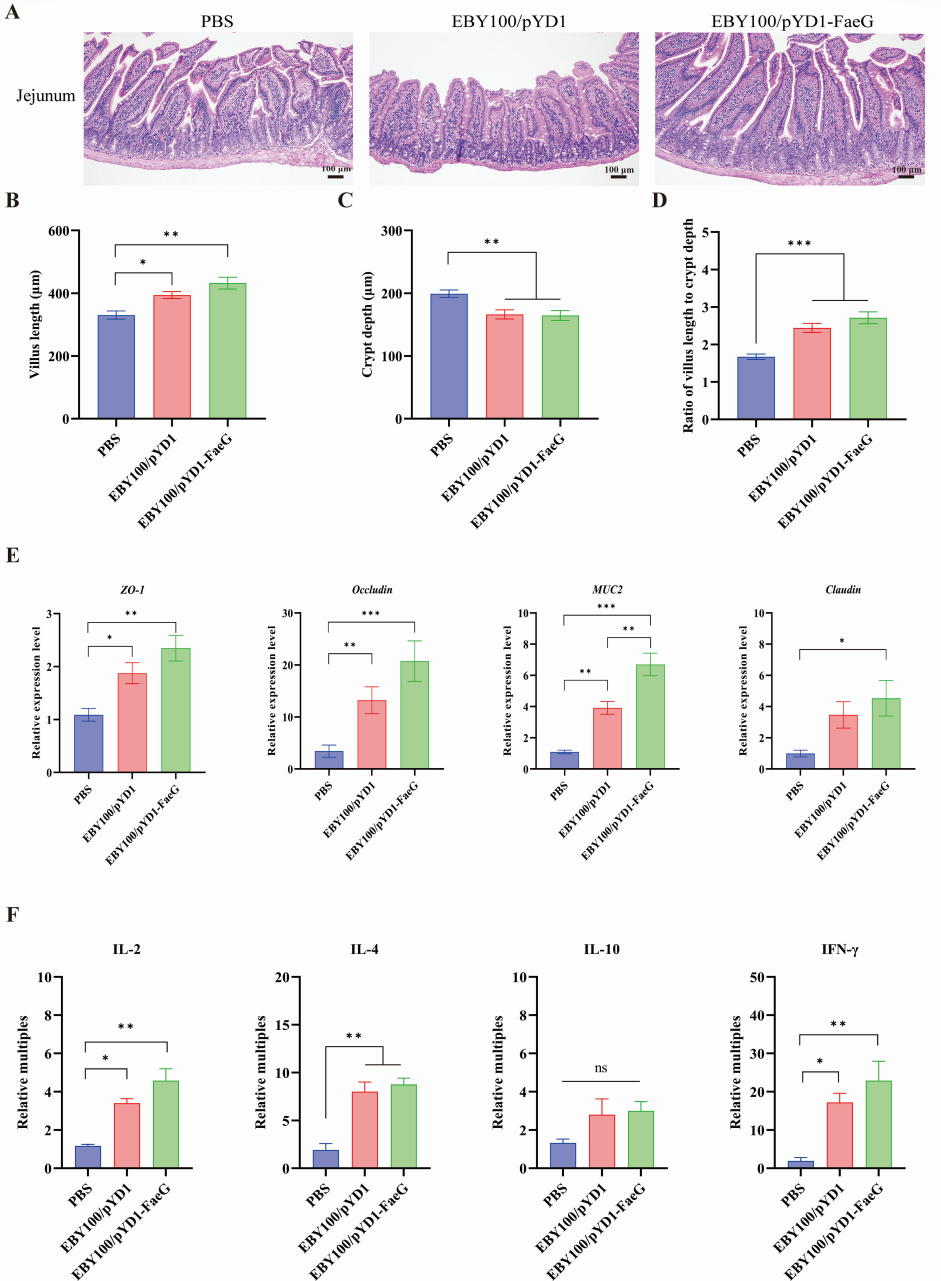
Effects of the EBY100/pYD1-FaeG strain on the intestinal tract of mice following oral immunization. (**A**) The histopathological changes in the jejunum of mice in different immunization groups after H-E staining. The bar represents 100 µm. Quantitative analysis of villus length (**B**) and crypt depth (**C**) in the small intestine of mice (*n* = 3). Six intestinal villi and crypts were taken from each section for measurement. (**D**) The ratio of villus length to crypt depth (the V/C ratio) in mice. (**E**) Effect of immunization of mice on the expression of tight junction proteins in the small intestine. The relative expression of the tight junction proteins *ZO-1*, *Occludin*, *MUC2*, and *Claudin* in the small intestine after immunization was detected by quantitative real-time PCR. (**F**) The expression levels of cytokines (IL-2, IL-4, IL-10, and IFN-γ) in the intestines of mice after immunization. The experiments were repeated three times, and the data in the figure are represented as mean ± SEM. The significance of the difference between the data in each group was determined by the *t*-test and the one-way ANOVA (*P < 0.05; **P < 0.01; and ***P < 0.001).

To examine the effect of the EBY100/pYD1-FaeG oral administration on intestinal mucosal immunity in mice, we observed the histological structure of the small intestine and assessed the expression levels of cytokines and tight junction proteins after immunization. The EBY100/pYD1 and EBY100/pYD1-FaeG groups showed significantly longer villi and shallower crypts compared to the PBS group (*P* < 0.05). Consequently, the V/C ratio (the villus length to the crypt depth) in the small intestine of these groups was substantially greater than that of the PBS group (*P* < 0.05, Fig. 4A through D).

Furthermore, the expression of tight junction proteins *ZO-1*, *Occludin*, *MUC2*, and *Claudin* in the small intestine from both the EBY100/pYD1 and EBY100/pYD1-FaeG groups significantly increased compared with the PBS group (*P* < 0.05, Fig. 4E). These results showed that immunization with the recombinant *S. cerevisiae* EBY100/pYD1-FaeG enhanced the integrity of the intestinal barrier.

Additionally, the results of quantitative real-time PCR (qPCR) detection of cytokine mRNA expression levels in the small intestine revealed that the EBY100/pYD1-FaeG strain significantly upregulated the expression levels of IL-2, IL-4, and IFN-γ in the intestinal mucosa compared with the PBS group (*P* < 0.05, Fig. 4F). Although the extent of cytokine upregulation observed with the EBY100/pYD1-FaeG group was greater than that of the EBY100/pYD1 group, this difference did not reach statistical significance (Fig. 4F). The experimental results showed that the recombinant *S. cerevisiae* EBY100/pYD1-FaeG has the potential to stimulate the intestinal mucosal immune response, thereby promoting both cellular and humoral immunity.

### Effects of the recombinant oral vaccine on gut microbiota composition in mice

**Fig 5:**
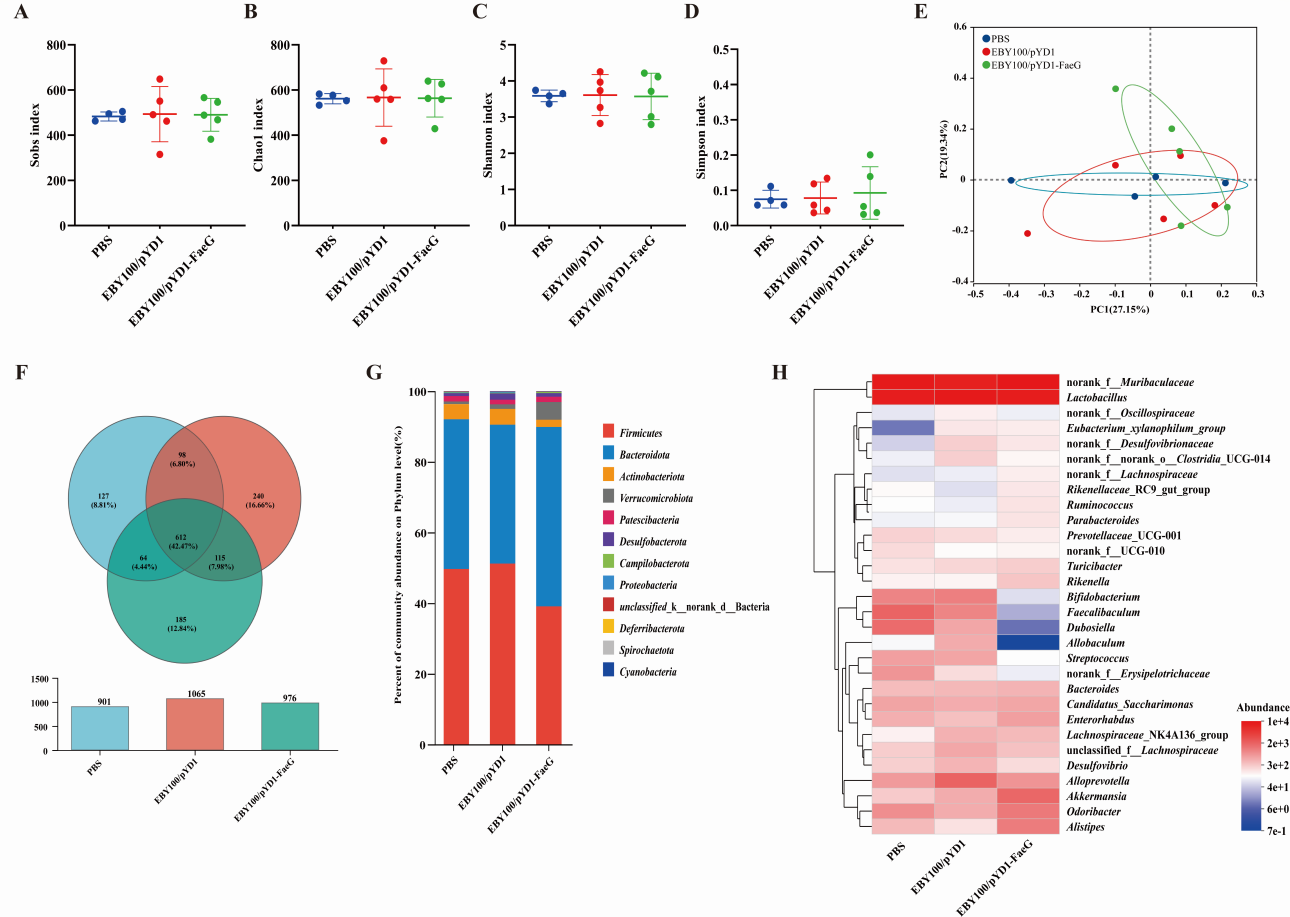
Effects of oral immunization with the recombinant *S. cerevisiae* strain EBY100/pYD1-FaeG on gut microbiota in mice. α-diversity of gut microbiota among the groups of mice after immunization is shown by Sobs (**A**), Chao1 (**B**), Shannon (**C**), and Simpson indices (**D**). (**E**) The PCoA plots show the β-diversity of gut microbial composition based on Bray-Curtis distance at the OTU level. (**F**) The Venn diagram shows the unique or shared OTUs in different samples. (**G**) Histogram of differences in species distribution of gut microbiota across phyla in each group of mice. (**H**) At the genus level (top 30 species), mouse gut microbial community composition was different. Data are shown as mean ± SEM (*n* = 5).

To investigate the impact of the recombinant EBY100/pYD1-FaeG strain on the gut microbiota composition in mice, the analysis of gut microbiota diversity was conducted using the Majorbio Cloud online platform following 16S rRNA sequencing. The α-diversity of the gut microbiota was assessed through various indices, including Sobs, Chao 1, Shannon, and Simpson, at the operational taxonomic unit (OTU) level, which reflects species abundance and diversity. The results of α-diversity indicated that the species richness and diversity of gut microbiota were comparable among the EBY100/pYD1-FaeG group, the EBY100/pYD1 group, and the PBS group, with no significant differences (Fig. 5A through D). In terms of β-diversity, which illustrates the similarities and differences in gut microbiota across different groups, the principal coordinates analysis (PCoA) revealed notable similarities in community composition between the PBS group and the EBY100/pYD1 group. Conversely, the EBY100/pYD1-FaeG group was different from both groups (Fig. 5E). These results suggested that the immunization of mice with the recombinant strain EBY100/pYD1-FaeG had a certain influence on gut microbiota.

A shared OTU analysis was used to further evaluate the similarities and differences in intestinal microbiota composition among different groups post-immunization. The analysis revealed that 612 OTUs were common among the intestinal microbiota of the three groups in mice post-immunization (Fig. 5F). Within the EBY100/pYD1-FaeG group, a total of 976 OTUs were identified, of which 185 were unique. The unique OTUs within the EBY100/pYD1-FaeG group, the EBY100/pYD1 group, and the PBS group constituted 12.84%, 16.66%, and 8.81% of their respective total OTUs (Fig. 5F).

To further investigate the variations in species composition across different groups, the changes in dominant species composition in different groups were analyzed at the phylum level (Fig. 5G). The highest relative abundance of *Firmicutes* in the gut microbiota of control groups (the PBS group and the EBY100/pYD1 group) reached values of 49.67% and 51.17%, respectively. *Bacteroidetes* occupied the second position in terms of relative abundance, comprising 42.38% and 39.36% of the microbiota in these groups, while *Actinobacteria* ranked third with relative abundances of 4.25% and 4.43% (Fig. 5G). In contrast, within the EBY100/pYD1-FaeG group, *Bacteroidetes* exhibited the highest relative abundance at 50.79%, followed by *Firmicutes* at 39.07%. Notably, the relative abundances of *Actinobacteria* decreased, whereas that of *Verrucomicrobia* increased to 5.03% (Fig. 5G). Furthermore, *Akkermansia* and *Odoribacter* showed significantly higher abundances in the EBY100/pYD1-FaeG group compared to the control groups at the genus level (Fig. 5H). Conversely, *Faecalibaculum*, *Bifidobacterium*, *Allobaculum*, and *Dubosiella* were significantly less abundant in the EBY100/pYD1-FaeG group relative to the control groups (Fig. 5H).

### The protective effects of the recombinant oral vaccine in mice

**Fig 6:**
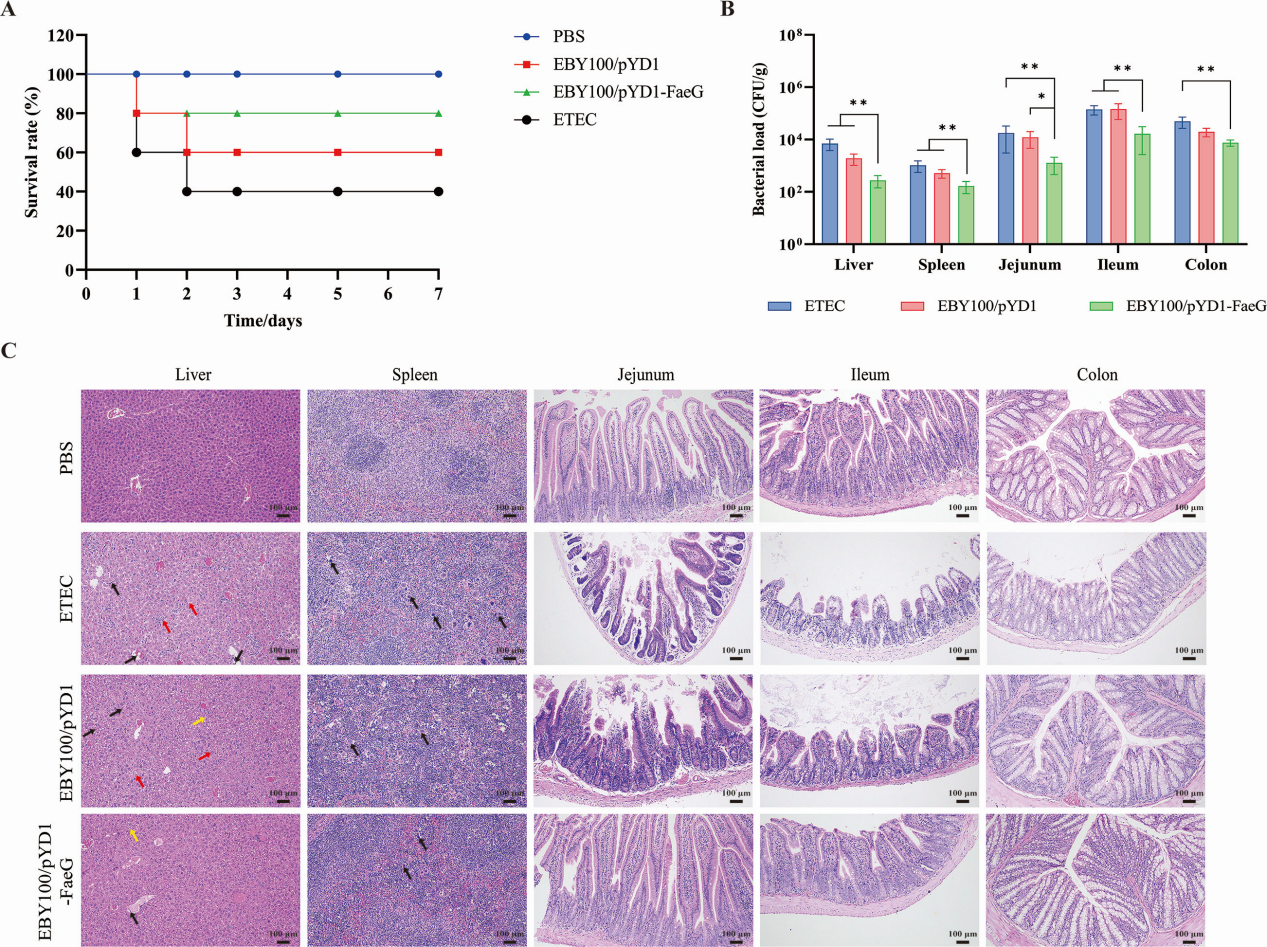
Immunoprotective effect of the recombinant strain on mice against ETEC EC6 infection after oral immunization. (**A**) Survival curve. On day 28 post-immunization, mice were challenged with LD_50_ of ETEC EC6 by i.p. (10^8^ CFU, 200 µL/mouse) and continuously monitored for 7 days. (**B**) Bacterial load in tissues and organs of mice 7 days post-infection. (**C**) Observation of histopathological changes in mouse tissues and organs 7 days post-infection with ETEC EC6. The pathological changes in liver, spleen, jejunum, ileum, and colon were observed by H-E staining. The black arrow points to inflammatory cells; the yellow arrow indicates cytoplasmic vacuolization; and the red arrow points to nuclear fragmentation. The bar represents 100 µm. All data were obtained from three independent replicates and presented as mean ± SEM. Statistical analysis was performed by *t*-test and one-way ANOVA (*P < 0.05 and **P < 0.01).

To assess the immune protection provided by the EBY100/pYD1-FaeG strain on mice following oral immunization, a random selection of 10 mice from each experimental group was conducted. These mice were subsequently challenged with F4+ ETEC EC6 of LD_50_ (200 µL, 10^8^ CFU) 7 days post-last immunization. Survival curves were plotted based on continuous monitoring of mortality over a period of 7 days (Fig. 6A). The findings indicated that the recombinant *S. cerevisiae* strain EBY100/pYD1-FaeG may have a good immune protection effect on mice after oral administration.

At 7 days post-challenge, the bacterial loads in the tissues and organs of the mice were measured. The ETEC EC6 loads in the livers, spleen, jejunum, ileum, and colon of the EBY100/pYD1-FaeG group were substantially reduced compared to the control groups (ETEC challenge group and EBY100/pYD1 group) (Fig. 6B). These results demonstrated that oral immunization utilizing the recombinant *S. cerevisiae* EBY100/pYD1-FaeG led to a decrease in the bacterial loads of ETEC EC6 within the tissues and organs of mice.

Furthermore, to comprehensively assess the protective efficacy of EBY100/pYD1-FaeG on various tissues and organs, the histopathological alterations in the liver, spleen, jejunum, ileum, and colon were examined through H-E staining in mice. As shown in Fig. 6C, the EBY100/pYD1-FaeG group significantly alleviated the pathological damage inflicted by F4+ ETEC EC6 on tissues and organs when compared to the ETEC group. The inflammatory cell infiltration, cytoplasmic vacuolization, and nuclear fragmentation were detected in the livers of mice infected with F4+ ETEC EC6. Additionally, the spleens of both the ETEC and EBY100/pYD1 groups were observed to have germinal centers that were not obvious, the white pulp and red pulp were structurally disordered without obvious boundaries, the lymphocyte count was significantly decreased, and the infiltration of inflammatory cells was severe. Conversely, in the EBY100/pYD1-FaeG group, the pathological damage of the spleen was significantly reduced, with discernible boundaries between the red and white pulp and a reduction in inflammatory cell infiltration (Fig. 6C).

In both the ETEC and EBY100/pYD1 groups, the jejunal villi exhibited disorganization characterized by distortion and lodging, significant shortening, and extensive shedding, and the structure of crypts was compromised, resulting in a reduced quantity (Fig. 6C). Furthermore, in the ileum, there was an observable widening of the gap between the villous epithelium and the lamina propria, and the lamina propria was hemorrhaged in the ETEC and EBY100/pYD1 groups (Fig. 6C). Conversely, in the EBY100/pYD1-FaeG group, the intestinal mucosal damage was significantly improved, the villus structure was relatively complete, and there was no obvious bleeding in the lamina propria (Fig. 6C).

These findings suggested that the recombinant *S. cerevisiae* strain EBY100/pYD1-FaeG has the potential to mitigate the pathological damage in mice following F4+ ETEC EC6 challenge. Additionally, these results indicate that the EBY100/pYD1-FaeG strain may serve as a viable candidate for a subunit vaccine aimed at controlling F4+ ETEC infection in the clinical settings.

## DISCUSSION

The antibiotic resistance of ETEC has been recognized as a significant contributor to the global increase in antibiotic resistance (33, 34). The inappropriate use of antibiotics on a global scale, particularly in livestock farming, has resulted in the widespread of resistant strains. This phenomenon presents a considerable risk to public health and adversely affects agricultural productivity. Consequently, the formulation of viable vaccines against ETEC will effectively reduce the reliance on antibiotics, thereby delaying the development of antibiotic-resistant strains. It will also effectively reduce the morbidity and mortality of PWD and greatly reduce the economic loss of the livestock breeding industry.

In the development of ETEC vaccines in humans and animals, candidate vaccines based on the expression of colonization factors have great potential (35, 36). At present, the PWD vaccines against ETEC infections in livestock primarily focus on the epidemic colonization factors, such as F4, F5, F6, and F41 (37, 38). These vaccines are commonly used to vaccinate sows before parturition to stimulate the production of specific IgG and IgA antibodies in colostrum, thereby providing some passive immune protection to newborn piglets (39). However, this approach mainly stimulates the systemic immune system rather than mucosal immunity in piglets and thus cannot completely prevent PWD infection (40).

In contrast, oral vaccines have the capacity to induce both systemic and mucosal immune responses, thereby facilitating the development of more extensive and long-lasting immunity (41). Oral vaccines can stimulate the mucosal production of sIgA, which is crucial for mucosal immunity and can effectively prevent pathogen colonization on the mucosal surface (42). Furthermore, oral vaccines have several advantages over injectable vaccines, such as better risk reduction of disease transmission, simple immunization, increased patient compliance, simple large-scale production, and more convenient storage and transportation (43, 44). *S. cerevisiae* not only possesses the ability to express antigens efficiently but also has adjuvant functions that can significantly enhance mucosal immune responses (22, 45).

Therefore, this study utilized *S. cerevisiae* EBY100 as a vector to express the FaeG protein, and the successful construction of the recombinant EBY100/pYD1-FaeG strain was confirmed through IFA and WB analysis. Oral administration of the EBY100/pYD1-FaeG vaccine directly stimulated the production of higher levels of IgG in serum and sIgA in the feces, while also improving the expression of IL-2, IL-4, and IFN-γ in the intestine. These findings provide additional evidence that oral vaccines can effectively stimulate intestinal mucosal immunity and play a strong protective role in preventing microbial infections (41). Additionally, after the challenge, the mortality rate of mice and the bacterial loads in various tissues were significantly reduced. These results further prove that oral vaccine immunization can stimulate the mouse body to produce peripheral immunity and mucosal immunity, thereby resisting ETEC EC6 infection and providing good protection for mice.

In addition, several research investigations have employed various probiotics as delivery systems for the expression of the FaeG protein to develop oral vaccines, and these research have also indicated the potential efficacy of the FaeG protein in combating F4+ ETEC infections (46–49). For instance, Yu et al. (48, 50) and Yang et al. (47) utilized *Lactococcus lactis, Lactobacillus casei,* and *Lactobacillus plantarum* as carriers in their research to express the FaeG fusion protein linked to immune-enhancing antigen fragments. Their findings indicated that these vaccines effectively elicited immune responses and provided protection against F4+ ETEC infection. Although these probiotic carriers have shown potential in ETEC vaccine development, the *S. cerevisiae* vaccine examined in this work demonstrated the capability to induce a robust mucosal immune response without requiring fusion with immune-enhancing antigen fragments. This may be attributed to the superior adjuvant characteristics and immunostimulatory capabilities of the *S. cerevisiae* carrier (45).

The research by Aves et al. (40) demonstrated that a vaccine based on highly immunogenic capsid virus-like particles displaying the FaeG protein induced significant systemic and mucosal antibody responses in mice and triggered a strong immune response in sows. Nonetheless, this strategy faced significant challenges due to interference from maternal antibodies, which impeded effective protection in piglets. Additionally, the methodology is relatively intricate, necessitating stringent standards for protein expression and purification, and it presents challenges for antigens that are difficult to express. In contrast, *S. cerevisiae* presents several advantages, including high expression levels, ease of genetic manipulation, straightforward cultivation, compatibility with large-scale fermentation, and cost-effectiveness, rendering it a more practical option for widespread production applications. Although *S. cerevisiae* presents numerous benefits as a vaccine carrier, further optimization is required for its large-scale production. In this study, we used an inducible promoter to drive antigen expression. This inducible system facilitates the control and optimization of both the timing and level of antigen expression to efficiently stimulate the immune response. However, compared with the constitutive promoter, which can be stably expressed without induction conditions, the inducible system may lack expression stability and immune persistence in the inducer within the gastrointestinal tract. This limitation also identifies important directions for our future research.

Gut microbiota represents a critical biological entity essential for maintaining health, and its composition and diversity directly affect the development and function of the host immune system from birth (51, 52). Several studies have demonstrated that yeast, as a probiotic, can enhance diversity and regulate the gut microbiota composition, subsequently improving intestinal immune function (24, 53, 54). Furthermore, the gut microbial composition can influence the efficacy of vaccinations, while changes in the immune status after vaccination may also affect gut microbial composition (27, 55, 56). This study revealed that the EBY100/pYD1-FaeG post-vaccination, there were significant changes in the intestinal flora structure, characterized by an increased relative abundance of *Bacteroidetes* and *Verrucomicrobia*, as well as enhanced species-level diversity in mice (Fig. 5). These results stand in contrast to those observed in the control groups, thereby underscoring the influence of vaccination on the composition of the intestinal microbiota in mice.

The villus length and crypt depth are widely recognized indicators for assessing intestinal health, and the crypt depth is inversely proportional to the rate of villus cell formation and the absorptive capacity of the villus (57). The shallower crypts indicate an increased rate of cell maturation and a stronger secretory function. In the study, we observed that the villus length increased, the crypt depth decreased, and the V/C ratio increased in the small intestine of mice following immunization with the EBY100/pYD1-FaeG. These findings indicated that oral vaccination may enhance nutrient absorption within the intestine.

Furthermore, tight junctions play a crucial role in preserving the integrity of the intestinal barrier (58–60). In our research, the increased relative expression of intestinal tight junction proteins (*ZO-1*, *Occludin,* and *MUC2*) further substantiates the hypothesis that oral immunization with the recombinant strain EBY100/pYD1-FaeG may strengthen the integrity of intestinal barrier function and contribute positively to the defense against ETEC EC6 infection.

The recombinant yeast EBY100/pYD1-FaeG has exhibited notable immunoprotective properties as an oral vaccine, particularly in its ability to stimulate mucosal immunity and enhance intestinal health. However, future research still needs to optimize the stability of the oral vaccine, the immunization dosage and protocols, as well as investigate its applicability across various animal populations.

### Conclusion

In summary, the recombinant *S. cerevisiae* strain EBY100/pYD1-FaeG expressing FaeG protein was successfully constructed. The live strains induced significant antibody levels of IgG and IgA by oral administration in mice, provided a good protective effect in the challenge experiment in mice, alleviated the pathological damage caused by ECET, and moderately decreased the bacterial load in the tissues and organs. These findings indicated that oral immunization with EBY100/pYD1-FaeG can effectively prevent F4+ ETEC infection, which could be used as a viable approach for preventing and controlling F4+ ETEC bacterial infection and provide a candidate for F4+ ETEC vaccine development.

## MATERIALS AND METHODS

### Strain and plasmid

The bacterial strain F4+ ETEC EC6 was stored in our laboratory (61). The *S. cerevisiae* EBY100 chemically competent cell was bought from Protein Interaction Bio. Co., Ltd (Wuhan, China). The *S. cerevisiae* EBY100 was grown in YPD medium (containing 20 g/L tryptone, 10 g/L yeast cell extract, and 20 g/L glucose or galactose). The plasmid pYD1 was bought from Hunan Fenghui Biotechnology Co., Ltd (Hunan, China).

### Recombinant plasmid construction

The FaeG gene (GenBank: DQ307494.1) was amplified using primers (FaeG-F: 5′-cgGCTAGCatgaaaaagactctgattgcactg-3′, FaeG-R: 5′-cgACGCGTgtaataagtaattgctacgttcagcg-3′) after it had been synthesized by Tsingke Biotechnology Co., Ltd. (Beijing, China), in which the capital letters represent the restriction enzyme sites *Nhe*I and *Mlu*I. The purified FaeG gene fragment was recombined into the plasmid pYD1 by T4 DNA ligase (Takara, Japan). Then, the recombinant plasmid pYD1-FaeG was purified in quantity from *E. coli* DH5α.

### Construction of the recombinant *S. cerevisiae* strains

The purified pYD1-FaeG plasmid was transformed into *S. cerevisiae* EBY100 competent cell using the LiAc method, as described in the instruction manual. Meanwhile, the empty pYD1 plasmid was also transformed into the *S. cerevisiae* EBY100 competent cell. The EBY100/pYD1-FaeG was screened using the minimal dextrose plates (containing 6.7 g/L YNB, 15 g/L agar, 20 g/L glucose, and 0.01% leucine) at 30°C. A single clone of EBY100/pYD1-FaeG was sequenced by amplification with primers (pYD1-F: 5′-AGTAACGTTTGTCAGTAATTGC-3′, pYD1-R: 5′- GTCGATTTTGTTACATCTACAC-3′) to verify the accurate recombination of the FaeG gene. It was selected into 4 mL YNB-CAA with glucose medium (containing 6.7 g/L YNB, 20 g/L glucose, and 5 g/L casamino acids) and then incubated at 30°C with shaking at 250 rpm until the absorbance of cell culture was about 2.0 at OD_600nm_. Finally, the cell pellet was centrifuged at 1,500 × *g* for 2 min, then added with YNB-CAA with galactose medium (containing 6.7 g/L YNB, 20 g/L galactose, and 5 g/L casamino acids) to OD_600nm_ of 0.8, and cultured at 20°C with shaking at 250 rpm for 72 h.

### Western blotting assay

The expression of the FaeG protein was confirmed using western blotting. Briefly, 1 mL of the EBY100/pYD1-FaeG culture was harvested for 48 h and washed with an equal volume of 1× PBS containing 50 mM tris-HCl (pH 7.5). Then, the cell pellets were suspended in 100 µL of ESB buffer (2% SDS, 1.5% DTT, 10% glycerin, 0.01% bromophenol blue, and 80 mM Tris-HCl, pH 6.8), boiled for 5 min, and frozen at −80°C for 5 min. The supernatant from the EBY100/pYD1-FaeG cell wall lysates was separated with 12% SDS-PAGE gel (Bio-Rad, USA) and transferred to a methanol-activated PVDF membrane (Bio-Rad, USA). After blocking with 5% skim milk (BioFroxx, China) for 2 h, the membrane was incubated with 6×His-tag antibody (Proteintech, USA) at room temperature for 1 h. It was subsequently incubated with an HRP-conjugated IgG antibody (Boster, China) for 45 min. Finally, the blot was visualized using BeyoECL Plus (Beyotime Biotechnology, Shanghai, China) and imaged using the Tanon Imager System (Tanon, Shanghai, China).

### Immunofluorescence assay and flow cytometric analysis

The cell pellets of 10^7^ CFU EBY100/pYD1-FaeG were washed twice with cold 1× PBS buffer and centrifuged at 1,500 × *g* for 2 min. The cell pellets were incubated with 200 µL of 1 µg/mL 6×His-tag primary antibody (containing 1 mg/mL BSA) on ice for 30 min and washed with cold 1× PBS buffer. The cells were incubated with 200 µL of 1 µg/mL IgG (H + L) and Alexa Fluor 488 (Invitrogen, USA) on ice for 30 min in the dark. After washing twice, 1 mL of PBS was added to the cell pellets to resuspend them, and they were analyzed using the CytoFLEX-LX flow cytometer (Beckman, USA) and FlowJo 10.8.1 software. Finally, a 10 µL solution was spread on a slide for immunofluorescence observation using a positive fluorescence microscope (Olympus, Japan).

### The growth curve and the expression kinetics of the EBY100/pYD1-FaeG strain

The strains EBY100/pYD1-FaeG, EBY100/pYD1, and EBY100 were grown in YPD medium with galactose at 30°C with shaking at 250 rpm for 60 h. The OD_600nm_ absorbance and the number of colonies on the YPD plates with galactose of the strains were measured at 0, 6, 12, 24, 30, 36, 48, and 60 h. The recombinant strain EBY100/pYD1-FaeG was resuspended in YNB-CAA with galactose medium to achieve an initial concentration of 0.5 OD_600nm_, then induced expression at 20°C with shaking at 250 rpm for 72 h. The 1 mL culture was taken at 0, 6, 12, 24, 30, 36, 48, 60, and 72 h, and the absorbance of the culture was adjusted to achieve an OD_600nm_ of 0.5. Then, the cell wall protein was extracted according to the method mentioned above (method 4), and the EBY100 cultured for 48 h was used as a negative control. Then, 20 µL supernatant of the cell wall lysates was tested via western blotting, according to above-mentioned method 4. Concurrently, an incubation of GAPDH antibodies was conducted on the PVDF membrane. Then, the blot was visualized and imaged after the secondary antibody incubation.

### Immunization and challenge experiment

The female SPF ICR mice, at 6 weeks of age, were randomly assigned to three distinct groups (15/group): (i) the EBY100/pYD1-FaeG group, (ii) the empty plasmid vector (EBY100/pYD1) group, and (iii) the PBS control group. The mice were orally administrated 200 µL of EBY100/pYD1(10^8^ CFU), EBY100/pYD1-FaeG (10^8^ CFU), and PBS for prime immunization and booster immunization on days 1–7 and 15–21, respectively.

In each group, the body weights of mice were recorded on days 7, 14, 21, and 28. In addition, the blood and feces samples of mice were collected on days 7, 14, 21, and 28 after the initial immunization. The blood was kept at 4°C overnight. Following centrifugation at 1,000 × *g* for 20 min at 4°C, the sera were collected and stored at −80°C. The collected feces (0.2 g/group) were added to 2 mL saline solution and ground at 60 Hz for 2 min using the grinder. The supernatant was collected and stored at −20°C after centrifugation at 10,000 × *g* for 2 min. Additionally, the heart, liver, spleen, kidneys, and lungs of mice (*n* = 5) were collected and weighed on 28th day, after which the organ index was calculated using the following formula:


$$Organ\;index\;\%=\frac{Organ\;weight}{Body\;weight}\times 100\%.$$

Seven days post-last immunization, 10 mice of each group were randomly selected to challenge intraperitoneal injection with 200 µL ETEC EC6 of LD_50_ (10^8^ CFU). Subsequently, the mortality of the mice was documented daily, and a survival rate curve was generated for 7 days following the challenge. Furthermore, 7 days post-infection, blood, tissues, organs, and intestinal feces were collected from the surviving mice of each group. The sera were isolated from the blood and stored at −80°C. To assess the bacterial loads of tissues and organs, 1 mL of sterile PBS buffer was added to the weighed tissues and organs. Then, these samples were ground three times using a grinder at 4°C, 60 Hz for 60 s each, with an interval for 3 s. The mixtures were diluted by a 10-fold gradient, and then, 10 µL of each gradient dilution was spotted on the plates supplemented with spectinomycin (50 µg/mL) and incubated overnight at 37°C. Then, the number of bacteria EC6 was counted on the plates, and the bacterial load of tissues and organs was calculated.

### Assessment of the small intestine histomorphology

After being fixed in 4% paraformaldehyde, the samples of tissues and organs were paraffin-embedded, dried, and thinly sectioned. After that, the tissue sections were stained with H-E and scrutinized under a microscope for histological and morphological features. The villus length and crypt depth within the intestine were measured using Image J software, and the V/C ratio (the villus length to crypt depth) was computed.

### Analysis of gut microbiota

Five mice in each group were randomly chosen and euthanized to collect colorectal contents for the analysis of gut microbiota composition 7 days post-last immunization (it is noteworthy that due to an experimental error, only four samples from the PBS group were available for analysis). The 16s rRNA sequencing was outsourced by Shanghai Majorbio Bio-Pharm Tech Co., Ltd (Shanghai, China). The 16S rRNA V3–V4 variable regions were amplified by the following primers (388F: 5′-ACTCCTACGGGGGGCAG-3′, 806R: 5′-GACTACHVGGGTWTCTAAT-3′). Sequencing was conducted utilizing the Illumina MiSeq platform. After optimizing and assembling the raw reads, the sequences were subsequently clustered into OTUs using UPARSE (version 7.1). Subsequently, these OTUs were annotated with species information by comparison with the Greengenes Database (version 13.8). To assess the alpha diversity of the samples, Sobs, Chao 1, Shannon, and Simpson indices were utilized. In contrast, the beta diversity was determined through the calculation of Bray-Curtis distances using Qiime2 diversity software. Subsequently, the Bray-Curtis distances associated with beta diversity were visualized via PCoA. Additionally, the variations in microbial community composition across the groups were examined at the OTU, phylum, and genus levels, respectively. All data processing was conducted using the online platform Majorbio Cloud (https://www.majorbio.com/).

### ELISA

Specific IgG levels in serum and sIgA levels in fecal samples were measured using an indirect ELISA. First, 200 µL bacterial pellets of F4+ ETEC strain EC6 (10^6^ CFU/mL) was coated on microtiter 96-well plates (2 × 10^5^ CFU/well) and incubated overnight at 4°C. Then, the plates were blocked with 200 µL of 10 mg/mL BSA at 37°C for 2 h, followed by washing three times with PBST. The serum samples and fecal supernatant samples were diluted in a twofold gradient. Next, 100 µL of dilution was added to each well and incubated at 37°C for 1 h. After washing three times with PBST, the plates were incubated with 100 µL of HRP-goat anti-mouse IgG at a dilution of 1: 5,000 or HRP-goat anti-mouse IgA (Invitrogen, USA) at a dilution of 1: 2,000 at 37°C for 1 h. Subsequently, the plates were incubated in the dark at 37°C for 30 min after adding 50 µL of TMB substrate solution (Solarbio, Beijing, China) to each wall. The reaction was stopped by adding 50 µL of 2 M H_2_SO_4_ (Solarbio, China), and the OD_450nm_ of each well was measured using a microplate spectrophotometer (Thermo Electron Corporation, USA). The endpoint titers of antibody were expressed as the reciprocal of the highest dilution. The highest dilution was defined as the value of OD_450nm_ greater than twice the mean of the control group plus one standard deviation.

### Quantitative real-time PCR assay

The expression levels of cytokines and tight junction proteins in the small intestinal tissue of mice from each group post-immunization were assessed using qPCR. First, total RNA was extracted from the small intestine of mice 7 days post-immunization with TRIzol reagent (Aidlab, China). Two micrograms of this RNA was reverse transcribed into cDNA using the cDNA Synthesis SuperMix (Yeasen Biotech, China). Then, with a 10-fold dilution of cDNA serving as the template, the qPCR amplification was performed using the SYBR Green Mix (Yeasen Biotech, China). The qPCR was carried out in 10 µL of reaction volume with three amplification stages (stage 1: 95°C for 5 min, 40 cycles of stage 2: 95°C for 10 s, 60°C for 30 s, and stage 3: melt curve analysis). Each sample was repeated three times, and the data were processed using the 2^-ΔΔCT^ method. A comprehensive list of all primers utilized is provided in Table S1.

### Statistical analysis

All experiments were performed in triplicate, and the results are expressed as means ± SD or means ± SEM. Statistical analysis was performed with GraphPad Prism 9.5 software (San Diego, CA, USA) using *t*-tests, one-way ANOVA, and two-way ANOVA.

## SUPPLEMENTAL MATERIAL

Table S1 aem.01817-24-s0001.docx Sequences of primers used for qPCR.

ASM does not own the copyrights to Supplemental Material that may be linked to, or accessed through, an article. The authors have granted ASM a non-exclusive, world-wide license to publish the Supplemental Material files. Please contact the corresponding author directly for reuse.

## References

[1] Nagy B, Fekete PZ. 2005. Enterotoxigenic Escherichia coli in veterinary medicine. Int J Med Microbiol 295:443–454. doi:10.1016/j.ijmm.2005.07.003 16238018

[2] Kuhlmann FM, Laine RO, Afrin S, Nakajima R, Akhtar M, Vickers T, Parker K, Nizam NN, Grigura V, Goss CW, Felgner PL, Rasko DA, Qadri F, Fleckenstein JM. 2021. Contribution of noncanonical antigens to virulence and adaptive immunity in human infection with enterotoxigenic E. coli. Infect Immun 89:e00041-21. doi:10.1128/IAI.00041-21 33558320 PMC8091098

[3] Fratto A, Torricelli M, Sebastiani C, Ciullo M, Felici A, Biagetti M. 2024. Survey on resistance occurrence for F4^+^ and F18^+^ enterotoxigenic Escherichia coli (ETEC) among pigs reared in Central Italy regions. Vet Res Commun 48:1279–1284. doi:10.1007/s11259-023-10287-8 38175328

[4] Sun Y, Kim SW. 2017. Intestinal challenge with enterotoxigenic Escherichia coli in pigs, and nutritional intervention to prevent postweaning diarrhea. Anim Nutr 3:322–330. doi:10.1016/j.aninu.2017.10.001 29767133 PMC5941267

[5] Gresse R, Chaucheyras-Durand F, Fleury MA, Van de Wiele T, Forano E, Blanquet-Diot S. 2017. Gut microbiota dysbiosis in postweaning piglets: understanding the keys to health. Trends Microbiol 25:851–873. doi:10.1016/j.tim.2017.05.004 28602521

[6] Zhang Y, Tan P, Zhao Y, Ma X. 2022. Enterotoxigenic Escherichia coli: intestinal pathogenesis mechanisms and colonization resistance by gut microbiota. Gut Microbes 14:2055943. doi:10.1080/19490976.2022.2055943 35358002 PMC8973357

[7] Dubreuil JD, Isaacson RE, Schifferli DM. 2016. Animal enterotoxigenic Escherichia coli. EcoSal Plus 7. doi:10.1128/ecosalplus.ESP-0006-2016 PMC5123703 27735786

[8] Tsekouras N, Meletis E, Kostoulas P, Labronikou G, Athanasakopoulou Z, Christodoulopoulos G, Billinis C, Papatsiros VG. 2023. Detection of enterotoxigenic Escherichia coli and Clostridia in the aetiology of neonatal piglet diarrhoea: important factors for their prevention. Life (Basel) 13:1092. doi:10.3390/life13051092 37240738 PMC10223568

[9] Ryu JH, Kim S, Park J, Choi KS. 2020. Characterization of virulence genes in Escherichia coli strains isolated from pre-weaned calves in the Republic of Korea. Acta Vet Scand 62:45. doi:10.1186/s13028-020-00543-1 32819409 PMC7439630

[10] Duarte ME, Garavito-Duarte Y, Kim SW. 2023. Impacts of F18^+^ Escherichia coli on intestinal health of nursery pigs and dietary interventions. Animals (Basel) 13:2791. doi:10.3390/ani13172791 37685055 PMC10487041

[11] Luise D, Lauridsen C, Bosi P, Trevisi P. 2019. Methodology and application of Escherichia coli F4 and F18 encoding infection models in post-weaning pigs. J Anim Sci Biotechnol 10:53. doi:10.1186/s40104-019-0352-7 31210932 PMC6567477

[12] García V, Gambino M, Pedersen K, Haugegaard S, Olsen JE, Herrero-Fresno A. 2020. F4- and F18-positive enterotoxigenic Escherichia coli isolates from diarrhea of postweaning pigs: genomic characterization. Appl Environ Microbiol 86:e01913-20. doi:10.1128/AEM.01913-20 PMC7657637 32948526

[13] Xia P, Wu Y, Lian S, Quan G, Wang Y, Zhu G. 2021. Deletion of FaeG alleviated enterotoxigenic Escherichia coli F4ac-induced apoptosis in the intestine. AMB Express 11:44. doi:10.1186/s13568-021-01201-z 33738650 PMC7973317

[14] Xia P, Wang Y, Zhu C, Zou Y, Yang Y, Liu W, Hardwidge PR, Zhu G. 2016. Porcine aminopeptidase N binds to F4+ enterotoxigenic Escherichia coli fimbriae. Vet Res 47:24. doi:10.1186/s13567-016-0313-5 26857562 PMC4746772

[15] Lu T, Moxley RA, Zhang W. 2019. Mapping the neutralizing epitopes of enterotoxigenic Escherichia coli K88 (F4) fimbrial adhesin and major subunit FaeG. Appl Environ Microbiol 85:e00329-19. doi:10.1128/AEM.00329-19 30926730 PMC6532040

[16] Antimicrobial Resistance Collaborators. 2022. Global burden of bacterial antimicrobial resistance in 2019: a systematic analysis. Lancet 399:629–655. doi:10.1016/S0140-6736(21)02724-0 35065702 PMC8841637

[17] Ekhlas D, Sanjuán JMO, Manzanilla EG, Leonard FC, Argüello H, Burgess CM. 2023. Comparison of antimicrobial resistant Escherichia coli isolated from Irish commercial pig farms with and without zinc oxide and antimicrobial usage. Gut Pathog 15:8. doi:10.1186/s13099-023-00534-3 36829209 PMC9951511

[18] Morsing MK, Larsen I, Pedersen KS, Weber NR, Nielsen JP. 2022. Efficacy of neomycin dosing regimens for treating enterotoxigenic Escherichia coli-related post-weaning diarrhoea in a Danish nursery pig herd not using medicinal zinc oxide. Porcine Health Manag 8:46. doi:10.1186/s40813-022-00283-w 36333767 PMC9635141

[19] Canibe N, Højberg O, Kongsted H, Vodolazska D, Lauridsen C, Nielsen TS, Schönherz AA. 2022. Review on preventive measures to reduce post-weaning diarrhoea in piglets. Animals (Basel) 12:2585. doi:10.3390/ani12192585 36230326 PMC9558551

[20] Belda I, Ruiz J, Santos A, Van Wyk N, Pretorius IS. 2019. Saccharomyces cerevisiae. Trends Genet 35:956–957. doi:10.1016/j.tig.2019.08.009 31630852

[21] Vieira Gomes AM, Souza Carmo T, Silva Carvalho L, Mendonça Bahia F, Parachin NS. 2018. Comparison of yeasts as hosts for recombinant protein production. Microorganisms 6:38. doi:10.3390/microorganisms6020038 29710826 PMC6027275

[22] Tan Y, Chen L, Li K, Lou B, Liu Y, Liu Z. 2022. Yeast as carrier for drug delivery and vaccine construction. J Control Release 346:358–379. doi:10.1016/j.jconrel.2022.04.032 35483637

[23] Brown GD, Gordon S. 2003. Fungal beta-glucans and mammalian immunity. Immunity 19:311–315. doi:10.1016/s1074-7613(03)00233-4 14499107

[24] Rodríguez-Nogales A, Algieri F, Garrido-Mesa J, Vezza T, Utrilla MP, Chueca N, García F, Rodríguez-Cabezas ME, Gálvez J. 2018. Intestinal anti-inflammatory effect of the probiotic Saccharomyces boulardii in DSS-induced colitis in mice: impact on microRNAs expression and gut microbiota composition. J Nutr Biochem 61:129–139. doi:10.1016/j.jnutbio.2018.08.005 30236870

[25] Cherf GM, Cochran JR. 2015. Applications of yeast surface display for protein engineering. Methods Mol Biol 1319:155–175. doi:10.1007/978-1-4939-2748-7_8 26060074 PMC4544684

[26] Lei H, Xie B, Gao T, Cen Q, Ren Y. 2020. Yeast display platform technology to prepare oral vaccine against lethal H7N9 virus challenge in mice. Microb Cell Fact 19:53. doi:10.1186/s12934-020-01316-1 32122351 PMC7053147

[27] Cao H, Hua D, Zhang H, Zhang H, Liu N, Feng Z, Li H, Zhao B, Zhang L, Guo Y, Huang J, Zhang L. 2022. Oral immunization of recombinant Saccharomyces cerevisiae expressing fiber-2 of fowl adenovirus serotype 4 induces protective immunity against homologous infection. Vet Microbiol 271:109490. doi:10.1016/j.vetmic.2022.109490 35709627

[28] Park SM, Mo AY, Lim JG, Chung HJ, Kim TG, Kim KJ, Cho DH, Yang MS, Kim DH. 2007. Surface displayed expression of a neutralizing epitope of spike protein from a Korean strain of porcine epidemic diarrhea virus. Biotechnol Bioprocess Eng 12:690–695. doi:10.1007/BF02931087 32218674 PMC7090475

[29] Xing H, Zhu L, Wang P, Zhao G, Zhou Z, Yang Y, Zou H, Yan X. 2022. Display of receptor-binding domain of SARS-CoV-2 Spike protein variants on the Saccharomyces cerevisiae cell surface. Front Immunol 13:935573. doi:10.3389/fimmu.2022.935573 36032096 PMC9412237

[30] Gao T, Ren Y, Li S, Lu X, Lei H. 2021. Immune response induced by oral administration with a Saccharomyces cerevisiae-based SARS-CoV-2 vaccine in mice. Microb Cell Fact 20:95. doi:10.1186/s12934-021-01584-5 33952256 PMC8097247

[31] Gao S, Zuo W, Kang C, Zou Z, Zhang K, Qiu J, Shang X, Li J, Zhang Y, Zuo Q, Zhao Y, Jin M. 2024. Saccharomyces cerevisiae oral immunization in mice using multi-antigen of the African swine fever virus elicits a robust immune response. Front Immunol 15:1373656. doi:10.3389/fimmu.2024.1373656 38742108 PMC11089227

[32] Wang L, Yang M, Luo S, Yang G, Lu X, Lu J, Chen J. 2023. Oral vaccination of recombinant Saccharomyces cerevisiae expressing ORF132 induces protective immunity against cyprinid herpesvirus-2. Vaccines (Basel) 11:186. doi:10.3390/vaccines11010186 36680030 PMC9861155

[33] Collignon PJ, McEwen SA. 2019. One health-its importance in helping to better control antimicrobial resistance. Trop Med Infect Dis 4:22. doi:10.3390/tropicalmed4010022 30700019 PMC6473376

[34] García A, Fox JG. 2021. A one health perspective for defining and deciphering Escherichia coli pathogenic potential in multiple hosts. Comp Med 71:3–45. doi:10.30802/AALAS-CM-20-000054 33419487 PMC7898170

[35] Svennerholm AM, Lundgren A. 2023. Developments in oral enterotoxigenic Escherichia coli vaccines. Curr Opin Immunol 84:102372. doi:10.1016/j.coi.2023.102372 37523966

[36] Svennerholm AM, Lundgren A, Leach S, Akhtar M, Qadri F. 2021. Mucosal immune responses against an oral enterotoxigenic Escherichia coli vaccine evaluated in clinical trials. J Infect Dis 224:S821–S828. doi:10.1093/infdis/jiab475 34550392 PMC8687049

[37] Matías J, Berzosa M, Pastor Y, Irache JM, Gamazo C. 2017. Maternal vaccination. Immunization of sows during pregnancy against ETEC infections. Vaccines (Basel) 5:48. doi:10.3390/vaccines5040048 29211052 PMC5748614

[38] Dubreuil JD. 2021. Pig vaccination strategies based on enterotoxigenic Escherichia coli toxins. Braz J Microbiol 52:2499–2509. doi:10.1007/s42770-021-00567-3 34244980 PMC8270777

[39] Nguyet LTY, Ounjai P, Ngamwongsatit N, Kaeoket K. 2024. The immune response of pregnant sow after vaccination with crude fimbriae (F4) extracts vaccine and immunoprotection of nursery pig against pathogenic E. coli (F4^+^ETEC). Acta Trop 254:107173. doi:10.1016/j.actatropica.2024.107173 38503364

[40] Aves KL, Guerra PR, Fresno AH, Saraiva MMS, Cox E, Bækbo PJ, Nielsen MA, Sander AF, Olsen JE. 2023. A virus-like particle-based F4 enterotoxigenic Escherichia coli vaccine is inhibited by maternally derived antibodies in piglets but generates robust responses in sows. Pathogens 12:1388. doi:10.3390/pathogens12121388 38133272 PMC10745950

[41] Austriaco N. 2023. Yeast oral vaccines against infectious diseases. Front Microbiol 14:1150412. doi:10.3389/fmicb.2023.1150412 37138614 PMC10149678

[42] Jin Z, Gao S, Cui X, Sun D, Zhao K. 2019. Adjuvants and delivery systems based on polymeric nanoparticles for mucosal vaccines. Int J Pharm 572:118731. doi:10.1016/j.ijpharm.2019.118731 31669213

[43] Alotaibi BS, Buabeid M, Ibrahim NA, Kharaba ZJ, Ijaz M, Murtaza G. 2021. Recent strategies driving oral biologic administration. Expert Rev Vaccines 20:1587–1601. doi:10.1080/14760584.2021.1990044 34612121

[44] Creighton RL, Woodrow KA. 2019. Microneedle-mediated vaccine delivery to the oral mucosa. Adv Healthc Mater 8:e1801180. doi:10.1002/adhm.201801180 30537400 PMC6476557

[45] De Smet R, Allais L, Cuvelier CA. 2014. Recent advances in oral vaccine development: yeast-derived β-glucan particles. Hum Vaccin Immunother 10:1309–1318. doi:10.4161/hv.28166 24553259 PMC4896516

[46] Liu S, Li Y, Xu Z. 2013. Induction of specific immune responses in piglets by intramuscular immunization with fimbrial adhesin FaeG expressed in Lactococcus lactis. Res Vet Sci 95:130–136. doi:10.1016/j.rvsc.2013.03.004 23540979

[47] Yang G, Jiang Y, Tong P, Li C, Yang W, Hu J, Ye L, Gu W, Shi C, Shan B, Wang C. 2017. Alleviation of enterotoxigenic Escherichia coli challenge by recombinant Lactobacillus plantarum expressing a FaeG- and DC-targeting peptide fusion protein. Benef Microbes 8:379–391. doi:10.3920/BM2016.0116 28504575

[48] Yu J, Fu J, Liu H, Kang C, Wang Z, Jin Y, Wu S, Li T, Yang R, Jin M, Chen H, Wang X. 2024. Application of recombinant lactic acid bacteria (LAB) live vector oral vaccine in the prevention of F4+ enterotoxigenic Escherichia coli. Vaccines (Basel) 12:304. doi:10.3390/vaccines12030304 38543938 PMC10974401

[49] Ou B, Jiang B, Jin D, Yang Y, Zhang M, Zhang D, Zhao H, Xu M, Song H, Wu W, Chen M, Lu T, Huang J, Seo H, Garcia C, Zheng W, Guo W, Lu Y, Jiang Y, Yang S, Kaushik RS, Li X, Zhang W, Zhu G. 2020. Engineered recombinant Escherichia coli probiotic strains integrated with F4 and F18 fimbriae cluster genes in the chromosome and their assessment of immunogenic efficacy in vivo. ACS Synth Biol 9:412–426. doi:10.1021/acssynbio.9b00430 31944664

[50] Yu M, Qi R, Chen C, Yin J, Ma S, Shi W, Wu Y, Ge J, Jiang Y, Tang L, Xu Y, Li Y. 2017. Immunogenicity of recombinant Lactobacillus casei-expressing F4 (K88) fimbrial adhesin FaeG in conjunction with a heat-labile enterotoxin A (LTAK63) and heat-labile enterotoxin B (LTB) of enterotoxigenic Escherichia coli as an oral adjuvant in mice. J Appl Microbiol 122:506–515. doi:10.1111/jam.13352 27860074

[51] Hou K, Wu ZX, Chen XY, Wang JQ, Zhang D, Xiao C, Zhu D, Koya JB, Wei L, Li J, Chen ZS. 2022. Microbiota in health and diseases. Signal Transduct Target Ther 7:135. doi:10.1038/s41392-022-00974-4 35461318 PMC9034083

[52] Dominguez-Bello MG, Godoy-Vitorino F, Knight R, Blaser MJ. 2019. Role of the microbiome in human development. Gut 68:1108–1114. doi:10.1136/gutjnl-2018-317503 30670574 PMC6580755

[53] Kaźmierczak-Siedlecka K, Ruszkowski J, Fic M, Folwarski M, Makarewicz W. 2020. Saccharomyces boulardii CNCM I-745: a non-bacterial microorganism used as probiotic agent in supporting treatment of selected diseases. Curr Microbiol 77:1987–1996. doi:10.1007/s00284-020-02053-9 32472262 PMC7415030

[54] Zhou X, Liang L, Sun B, Li K, Guo H, Zhang Y. 2024. The effects of yeast protein on gut microbiota in mice when compared with soybean protein and whey protein isolates. Nutrients 16:458. doi:10.3390/nu16030458 38337742 PMC10857369

[55] Orso C, Stefanello TB, Franceschi CH, Mann MB, Varela APM, Castro IMS, Frazzon J, Frazzon APG, Andretta I, Ribeiro AML. 2021. Changes in the ceca microbiota of broilers vaccinated for coccidiosis or supplemented with salinomycin. Poult Sci 100:100969. doi:10.1016/j.psj.2020.12.066 33684651 PMC7938242

[56] Zhang L, Yao L, Guo Y, Li X, Ma L, Sun R, Han X, Liu J, Huang J. 2022. Oral SARS-CoV-2 spike protein recombinant yeast candidate prompts specific antibody and gut microbiota reconstruction in mice. Front Microbiol 13:792532. doi:10.3389/fmicb.2022.792532 35464985 PMC9022078

[57] Gehart H, Clevers H. 2019. Tales from the crypt: new insights into intestinal stem cells. Nat Rev Gastroenterol Hepatol 16:19–34. doi:10.1038/s41575-018-0081-y 30429586

[58] Suzuki T. 2020. Regulation of the intestinal barrier by nutrients: the role of tight junctions. Anim Sci J 91:e13357. doi:10.1111/asj.13357 32219956 PMC7187240

[59] Saito AC, Higashi T, Fukazawa Y, Otani T, Tauchi M, Higashi AY, Furuse M, Chiba H. 2021. Occludin and tricellulin facilitate formation of anastomosing tight-junction strand network to improve barrier function. Mol Biol Cell 32:722–738. doi:10.1091/mbc.E20-07-0464 33566640 PMC8108510

[60] Kuo W, Odenwald MA, Turner JR, Zuo L. 2022. Tight junction proteins occludin and ZO‐1 as regulators of epithelial proliferation and survival. Ann N Y Acad Sci 1514:21–33. doi:10.1111/nyas.14798 35580994 PMC9427709

[61] Hu D, Qian P, Gao D, Li X, Wang L, Ji H, Wang S, Li X. 2024. Characterization and genomics analysis of phage PGX1 against multidrug-resistant enterotoxigenic E. coli with in vivo and in vitro efficacy assessment. Anim Dis 4:7. doi:10.1186/s44149-024-00112-3

