# Supplementary material for: Recombinant Saccharomyces cerevisiae EBY100/pYD1-FaeG: a candidate for an oral subunit vaccine against F4+ ETEC infection
Source: Appl Environ Microbiol. 2024 Nov 27;91(1):e01817-24. doi: 10.1128/aem.01817-24 (PMC11784076; doi:10.1128/aem.01817-24)
Supplement: Table S1 — Sequences of primers used for qPCR. [file aem.01817-24-s0001.docx]

**Supplementary information:**

Table S1: Sequences of primers used for qPCR.

| **Gene** | **Sequence (5’–3’)** |
| --- | --- |
| *ZO-1* | F: CTGGTGAAGTCTCGGAAAAATG |
|  | R: CATCTCTTGCTGCCAAACTATC |
| *Occludin* | F: CAGGATGCCAATTACCATCAAG |
|  | R: GGGTTCACTCCCATTATGTACA |
| *MUC2* | F: CGAGCACATCACCTACCACATCATC |
|  | R: TCCAGAATCCAGCCAGCCAGTC |
| *Claudin* | F: AGATACAGTGCAAAGTCTTCGA |
|  | R: CAGGATGCCAATTACCATCAAG |
| IL-2 | F: CTCGGAGCTCTGCAGCGTGT |
|  | R: TCCACCACAGTTGCTGGCTCATC |
| IL-4 | F: CCACGGAGAACGAGCTCATC |
|  | R: GAGAACCCCAGACTTGTTCTTCA |
| IFN-γ | F: ACAACCCACAGATCCAGC |
|  | R: TCAGC ACCG ACTCCTTTT |
| TNF-α | F: CCACGCTCTTCTGTCTACTG |
|  | R: ACTTGGTGGTTTGCTACGA |
| β-Actin | F: TTGTCCCTGTATGCCTCTGG |
|  | R: ATGTCACGCACGATTTCCC |
